# Using colour in figures: let’s agree to differ

**DOI:** 10.1111/j.1600-0854.2008.00863.x

**Published:** 2008-12-04

**Authors:** Tim Levine

**Affiliations:** Department of Cell Biology, UCL Institute of OphthalmologyBath Street, London EC1V 9EL, UK

A picture can paint a thousand words, and cell biologists particularly rely on pictorial representations. It is now standard to use colour in pictures from graphs to micrographs. Apart from being appealing, colour can be justified on the basis that it adds information. However, a minority of people do not have the full range of colour vision (so-called ‘colour blind’), and this minority are too often excluded by ill-conceived use of colour. This article not only suggests ways to maximize the accessibility of colour images to the minority but also shows that many images in the field of membrane traffic cannot be viewed optimally by all, no matter what way they are treated.

**Figure 1:**
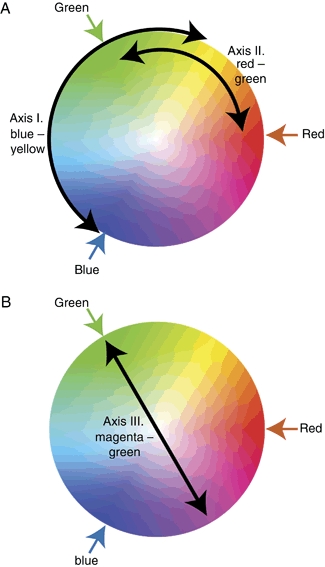
The human visual system is trichromatic but does not treat colours equally Hue and saturation are represented in a single two-dimensional colour wheel. Hue varies around the wheel, and saturation increases with distance from the centre. This colour wheel shows colours at maximum brightness (i.e. with no added black). Primary colours are indicated. A) The human central nervous system is trichromatic by applying two colour axes: I. Blue/yellow (through green) – which is ancient; II. Red/green (through yellow) – which is recent. We never experience yellowish blue, or greenish red; instead, we are equipped to perceive a spectrum of many hues along each axis. B) Digital screens are trichromatic treating the three primary colours equally in the RGB system. Red/green merges created *in silico*by cell biologists are similar to the naturally occurring red/green axis, with overlap perceived in a continuous spectrum of hues red↔yellow↔green. By comparison, axis III: magenta/green, which can be easily created *in silico*and which has been proposed to help the minority who lack a green or red opsin, uses only two hues: magenta and green and passes through the white (0% saturated) centre of the colour wheel. For the minority, the small amount of information in axis III is a distinct improvement over their inability to use axis II at all. However, the human brain is poorly equipped to assess degree of saturation, so the majority find axis III far less informative than axis II. The result of this is that no single treatment suits presentation of complex colour images to all people. Instead, different images must be treated according to the information they carry (Figures 2–4).

Colour vision is ancient in evolutionary terms, originally consisting of blue and yellow cones only (Figure 1A). In recent primate evolution, a duplication of the yellow opsin (on the X chromosome) has led to green–red discrimination. In terms of human cone numbers, this axis far outweighs the older one, with blue cones being relatively sparse. Thus, an individual with the full range of colour vision can accurately discriminate an entire spectrum of colour hues along the green–red axis better than along any other colour axis (for example green–blue). Note that humans differ considerably from digital cameras and video screens, where all colours are treated equally (Figure 1B). The paired green–red opsin genes often undergo partial or complete gene conversion leading to functional loss of green–red discrimination, again partial or complete. The incidence of the gene-converted haplotype is quite high in all racial groups; for example, 8% in Caucasians, where the incidence of the phenotype is 8% in males and 0.5% in females. The minority that lacks the full range of colour vision is commonly referred to as colour blind but almost all see some colour. Another argument against the common “colour blind” term is that it is politically undesirable to consider the genetic majority as better, particularly because ‘defective’ genes are the source of social discrimination in some countries. To avoid this, colour vision is here divided into the majority and minority types.

**Figure 2:**
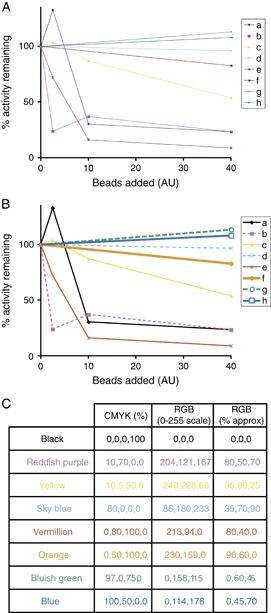
Some colours are more equal than others A and B) The two graphs show a single experiment relating to a pull down of an activity on beads. A) The graph uses the default settings provided by MicrosoftExcel™. B) The graph has been adjusted to make the data sets clearly identifiable. In general, diagrams should be designed so that if viewed in black and white they still work. Colour can add ornamentation but should not be essential. The most important changes are to enlarge symbols and thicken lines. When choosing colour, avoid pure red, green or blue and vary brightness as well as hue (Figure 1). Also, in legends, do not refer to data sets by their colour, which may not be recognizable by the minority. C) A palette of colours suggested by Masataka Okabe and Kei Ito (Tokyo), which are distinguishable by most of the minority with poor or no green–red discrimination.

There is good evidence that loss of the green–red axis is a balanced polymorphism, as under some circumstances the inability to distinguish green and red is beneficial (1). However, in cell biology presentations of the current day, it can cause considerable problems. So, how can the majority apply colour to their data without excluding the minority? Unfortunately, there is no quick fix, but with a moderate amount of thought, their results can be presented so that everyone can appreciate them. I have identified three categories of images that need to be treated differently.

Category 1 applies to all diagrams, including graphs (Figure 2). Simple adaptations help rapid processing of the information, with the minority colour vision types catered for by choosing the colour palette carefully. These rules should be applied in all walks of life as there is nothing specific to membrane traffic here. For example, the World Wide Web Consortium published in 1999 a set of Web Content Accessibility Guidelines (WCAG version 1.0) that have become the internationally accepted standard (www.w3.org/TR/WCAG10/). This includes simple advice on the use of colour.

Multiple micrographs of the same structures are the very stuff of membrane cell biology and particularly common in this journal. The common practice is to merge green and red images, which can produce a third channel (yellow) that (in very simplistic terms – see below) indicates the degree of overlap. But, as described above, people with the minority form of colour vision are specifically unable to access green–red merges. So, a solution should be sought for presenting these images to >99.9% (compared with 95%) of an audience. That something should be done is often agreed upon, for example in a recent spate of correspondence in Nature (2–4). However, no one has yet agreed on one simple algorithm. In my opinion, the solution lies in applying some intelligence – each presenter needs to understand a little bit about the problem and treat images according to their contents. Specifically, we need to make a judgement about the overlap being presented: is the overlap qualitative (important in a gross manner) or is it quantitative (important in fine detail)?

**Figure 3:**
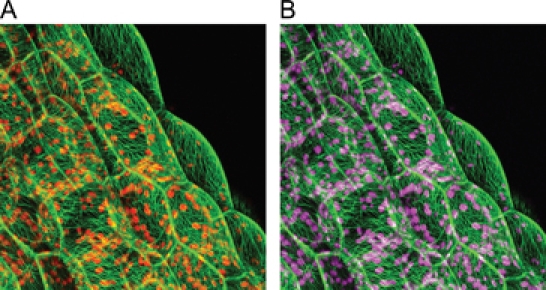
Simple two-colour micrographs where overlap is not crucial Images of Arabidopsis hypocotyl cells labelled for the chloroplasts and microtubules. A) Falsely coloured green/red, microtubules in green and chloroplasts in red. The two subcellular organelles sets landmarks for each other, and assessment of degree of overlap is not critical. Note that although the original fluorophores were similar to these colours (e.g. microtubules were decorated with GFP), the image could easily be shown with the colours reversed. B) A green/magenta image of the same data. With the image in RGB mode, all the information in the red channel was copied into the clipboard and pasted into the blue channel. The result is more informative for the minority, and does not reduce information for the majority, because the spectrum of hues on the green–red axis is not important. Image kindly provided by Juliet Coates (Birmingham, UK).

Until now, the green–red axis (which is impenetrable to the minority) has been used to present almost all merged images. I suggest that in future, this should be used only where strictly needed (see category 3 below). I suggest that category 2 images be considered all those simple two-colour images that do not need the full range of information offered by the green–red axis. The distinguishing feature of these images is that the two channels contain information of quite different types, for example two unrelated subcellular structures (Figure 3). Here, the areas of overlap are either not obvious at all, or relatively large and very obvious. The overlap is quantal (yes/no), and there is no need for a viewer to assess the precise degree of mixing between channels, so they do not require the subtleties afforded to the majority by the green–red axis. To present category 2 images, the most widely accessible merge is to have one channel green, the other magenta, with the merge colour being white (Figure 1B). This is very easily achieved when starting from green–red RGB images by pasting the information in the red channel into the (empty) blue channel (Figure 3B). If a third image of the same cells is also to be merged, as long as it shows similarly simple information, such as a nuclear counterstain, the third (blue) channel can be included in the merge. Here, instead of creating green–magenta merges, more complex algorithms may be used, for example the one linked to at http://www.vischeck.com/daltonize (Bob Dougherty, Stanford and Alex Wade, Smith-Kettlewell), which increases separation between green and red.

**Figure 4:**
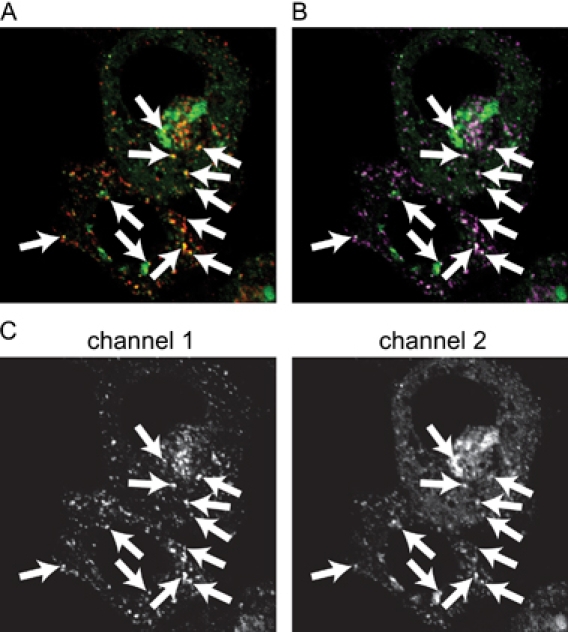
Complex two-colour micrographs where overlap is crucial Images of two markers with punctate distributions inside mammalian cells detected by immunofluorescence (kindly provided by Adam Grieve, UCL, London). A) Green/red merge – the standard method. B) The same data as a green/magenta merge. C) The two separate images in black and white. Arrows marking the most prominent double-positive puncta were created on one panel and placed identically on other panels using the ‘Align’ function (available in software such as AdobeIllustrator™ or MicrosoftPowerpoint™). While an image such as (A) contains maximal information for the majority, it is useless for the minority. Other images should be made available to allow the minority to assess overlap. B) The image does not use the trichromatic colour system of the majority to maximum advantage and is only partially useful to the minority for the same reasons. C) The image provides extra light, but the appreciation of overlap must be indirect. While this is not ideal, it does allow for careful (although non-intuitive) analysis, which is my preferred option for the minority.

What about images of highly overlapping data? Membrane cell biologists often produce images of two or three partially colocalizing punctate intracellular markers (Figure 4). Here, the extent of overlap is absolutely key. In these cases, green–red merges (Figure 4A), because they use the pathway that is by far the best-developed, are noticeably more informative than green–magenta images (Figure 4B). It would be a mistake for the minority to insist that the majority forego the highly useful and unique resource of green–red merges (2,4) because of their whole range of intermediate hues (Figure 1A). By comparison, green–magenta merges only have two hues, and the spectrum is created by varying saturation, where the visual system cannot easily distinguish intermediates (Figure 1B). In these instances, therefore, green–red merges should be preserved despite the fact that they are not readable by the minority.

For category 3, the minority must still be catered for, by showing the separate channels in individual greyscale images. Here, it is vital that the precise relationship between the two channels is indicated, usually by arrows placed identically on the two separate images (Figure 4C). As someone with minority colour vision, I can vouch for this approach. In a journal where printed space is lacking, these panels can be published as supplementary information online. In data presentations, be they to large conferences or to small lab meetings, the extra panels can be shown by toggling between the two images placed (and labelled with arrows) identically on successive slides. An important point about single channel images is that they should always be in black and white – never falsely coloured (which is often done solely to remind the reader of the colour used on a subsequent merge panel). This is because colours provide far less optical information than white. This applies especially when images are printed, when as much as 50% of the brightest pixels will be saturated with maximum ink levels.

Here, I have suggested a way to categorize and treat colour images so that close to 100% of people can access them, as opposed to 95%. Maybe, given the high proportion of category 3 images generated by the field of membrane traffic, our discipline should lead the way in defining standards for the use of colour in science and society at large. We should work to develop a consensus position that can be adopted by international journals and national/international scientific societies. At heart, as hinted at by the majority/minority terminology I have used, the question is political (very much with a small ‘p’). When you next present your work, are you prepared for 1 in 20 of your audience to not get the picture?
